# Attentional function in fibromyalgia and rheumatoid arthritis

**DOI:** 10.1371/journal.pone.0246128

**Published:** 2021-01-27

**Authors:** Carmen M. Galvez-Sánchez, Pablo de la Coba, José M. Colmenero, Gustavo A. Reyes del Paso, Stefan Duschek

**Affiliations:** 1 Department of Psychology, University of Jaén, Jaén, Spain; 2 Institute of Psychology, UMIT—University for Health Sciences, Medical Informatics and Technology, Hall in Tirol, Austria; University of Valencia, SPAIN

## Abstract

Concentration difficulties, forgetfulness and mental slowness are common in fibromyalgia syndrome (FMS); initial findings suggest that rheumatoid arthritis (RA) may also be accompanied by cognitive impairments. This study aimed to compare attentional performance between patients with FMS and RA. Attention was quantified in the domains of alerting, orienting and executive control using the Attentional Network Test–Interaction (ANT-I) in 56 women with FMS, 41 women with RA and 50 healthy women. Pain severity was statistically controlled in the group comparison. While FMS patients exhibited longer reaction times and made more errors on the ANT-I than RA patients and healthy women, performance did not differ between RA patients and healthy women. The magnitude of group differences did not vary by the experimental conditions of the ANT-I, suggesting a general attentional deficit in FMS rather than specific impairments in the domains of alerting, orienting and executive control. Differences between patient groups may relate to the different pathogenetic mechanisms involved in the disorders, i.e. inflammatory processes in RA and central nervous sensitization in FMS. In FMS, heightened activity in the pain neuromatrix may interfere with attention, because it requires enhanced neural resources in brain areas that are involved in both pain and attentional processing.

## Introduction

Fibromyalgia syndrome (FMS) is a chronic condition of widespread pain accompanied by complaints such as fatigue, sleep disturbance and symptoms of depression and anxiety [1]. In addition, there is evidence of cognitive problems in FMS, including deficits in attention, perception, memory and higher cognitive functions [2–4]. Though effect sizes differed considerably among studies, these alterations are in accordance with patients´ reports and significantly affect their everyday life and social and professional activities [2]. Cognitive complaints in FMS are frequently referred to as “fibro fog” [4, 5]; the term “dyscognition” is also used, which describes both subjective and objective cognitive impairments, including difficulties with basic and complex cognitive processes [6]. Between 50% and 90% of FMS patients report cognitive symptoms; their prevalence is higher than in other pain disorders [5]. Therefore, it has been suggested that routine screening for cognitive impairments in FMS patients should be included in FMS diagnostics [4].

Rheumatoid arthritis (RA) is a persistent autoimmune disorder mainly characterized by painful, stiff and swollen joints [7]. Cognitive problems may also occur in RA. Though the evidence is weaker than that for FMS, initial findings suggest impairments in the domains of attention, memory and verbal functions [8]. Moreover, deficits in visuospatial processes and mental flexibility were seen in RA [9]. At cerebral level, these deficits have been related to hypoperfusion of the frontal and parietal lobes and brain white matter alterations [9].

Studies directly comparing cognitive performance between FMS and RA are still scarce, but the available evidence suggests deficits of similar magnitude in these two patient groups [10–12]. Precisely, impairments in selective and sustained attention [10], working memory [11] and executive functions [12] did not differ between patients with FMS and RA. However, the sample sizes and statistical power of these studies were relatively low; as such, possible small or moderate differences in cognitive performance between patients’ groups may not have been detected in these studies [10–12]. The present study aimed at comprehensive assessment of attentional functions in FMS and RA; to obtain sufficient statistical power, appropriate sample size was á priori estimated based on effect sizes previously reported for attention deficits in FMS.

The comparison of cognitive performance between FMS and RA is of interest insofar as the pathogenetic mechanisms involved in the two syndromes are substantially different. While inflammatory processes underlie RA symptoms, central nervous factors are believed to play a key role in those of FMS. Neuroimaging studies suggested that FMS pain relates to central nervous sensitization and heightened activity in the pain neuromatrix [13]. It has been claimed that increased nociceptive activity interferes with cognition, because it requires greater neural resources in brain areas involved in both cognitive and pain processing [3, 14]. Not much is known about the mechanisms underlying the cognitive impairments seen in RA [9]. As an attention-demanding condition, pain may interfere with cognitive performance irrespective of nociceptive sensitization. Moreover, deficits might relate to psychiatric comorbidities or medication; in particular, the negative effects of pharmacologic therapy on cognition are recognized [8].

The present study investigated attentional performance in FMS and RA. The domain of attention was evaluated due to the frequent impairments seen therein in individuals with chronic pain, and the high relevance of attention to daily activities [3, 8]. Performance was assessed using the classical model of Posner and Petersen [15], which distinguishes three basic attentional subsystems: alerting, orienting, and executive control. The alerting system enables maintenance of an appropriate level of vigilance; orienting refers to the ability to select and prioritize relevant information from sensory input; and executive control refers to target detection and top-down supervisory control of information processing. In the study, patients with FMS and RA and healthy controls completed the Attentional Network Test (ANT-I) [16], which enables quantification of the three attentional subsystems. Considering possible influences of medication [5, 17] and affective alterations [2, 18, 19] on cognitive performance, effects of analgesic and psychotropic drugs and comorbid depression and anxiety disorders on attention were additionally investigated in patients. Finally, associations between clinical symptoms (pain, depression, anxiety, fatigue and insomnia) and attentional performance were quantified in both patient groups.

## Materials and methods

### Participants

In total, 56 women with FMS, 41 women with RA and 50 healthy women participated. Patients were recruited via the Fibromyalgia Association and Rheumatoid Arthritis Association of Jaén (Spain). All diagnoses were made by a rheumatologist according to the 2010 American College of Rheumatology criteria for FMS [1] or the 1987 American Rheumatology Association criteria for RA [20]. Only women aged between 30 and 65 years were allowed to participate. To ensure the comprehension of task´s instructions and questionnaires, elementary primary studies were additionally required. Patients with comorbid FMS and RA were excluded from the study. Exclusion criteria for all study groups were metabolic abnormalities, neurological disorders, drug abuse and other severe somatic (e.g., cancer) or psychiatric (e.g., psychotic) diseases. The control group was additionally required to be free from acute or chronic pain of any kind. Healthy participants were recruited from voluntary and neighborhood associations. Table 1 displays the demographic and clinical data of the sample.

**Table 1:** Sociodemographic data and questionnaire scores of the sample (M±SD); statistics of the group comparisons.

	FMS patients (N = 56)	RA patients (N = 41)	Control group (N = 50)	F[2, 144] / χ^2^	p	
**Age (years)**	52.39±7.74	51.95±10.48	51.18±6.57	0.29	.75	< .01
**Body mass index (kg/m**^**2**^**)**	27.60±4.85	27.96±6.19	26.46±2.67	1.33	.27	.02
**Duration of Education (years)**	9.95±4.28	9.90±4.14	11.54±3.92	2.53	.08	.03
**Depression (%)**^**a,b**^	38 (67.86%)	13 (31.71%)	5 (10%)	38.48	< .001	.51
Anxiety disorders** (%)^**b,c**^	31 (55.36%)	19 (46.34%)	6 (12%)	22.70	< .001	.39
**Antidepressant use (%)**^**a,b**^	47 (83.93%)	9 (21.95%)	5 (10%)	68.42	< .001	.68
**Anxiolytic use (%)**^**a,b**^	44 (78.57%)	11 (26.83%)	5 (10%)	56.02	< .001	.62
**Non-opioid analgesic use (%)**^**b,c**^	47 (83.93%)	30 (73.17%)	3 (6%)	72.73	< .001	.70
**Opiate use (%)**^**b,c**^	28 (50%)	12 (29.27%)	0 (0%)	33.46	< .001	.48
**Total Pain (MPQ)**^**a,b,c**^	81.23±35.53	44.56±27.06	12.82±21.44	73.75	< .001	.51
**State Anxiety (STAI-E)**^**a,b,c**^	23.30±4.25	18.78±13.39	13.02±10.16	15.23	< .001	.18
**Trait Anxiety (STAI-T)**^**a,b**^	51.50±9.61	30.59±14.56	27.32±17.85	44.71	< .001	.39
**Depression (BDI)** ^**a,b**^	44.14±13.71	13.29±15.60	11.40±15.06	81.58	< .001	.53
**Fatigue (FSS)** ^**a,b,c**^	52.52±9.73	33.37±18.54	23.40±15.00	55.85	< .001	.45
**Insomnia (COS)** ^**a,b**^	40.23±9.64	16.09±13.15	12.90±8.36	107.90	< .001	.60

Note: a = significant difference between FMS patients and RA patients,

b = significant difference between FMS patients and controls, and

c = significant difference between RA patients and controls.

* Anxiety disorders comprised panic disorder, generalized anxiety disorder, phobias and adjustment disorders.

### Instruments and measures

The patients´ clinical history and demographic data were obtained in a semi-structured interview. The Structured Clinical Interview for Axis I Disorders of the Diagnostic and Statistical Manual for Mental Disorders [SCID, 21] was applied to diagnose mental disorders. In addition, the following self-report questionnaires were administered:

- McGill Pain Questionnaire (MPQ) [22, 23]. This instrument includes 73 items to assess the sensorial, emotional and cognitive-evaluative components of pain experience. The total MPQ score (Total Pain, range: 0–146) was used in the study. A Cronbach´s α value of .74 was reported for this index [23].
- State-Trait Anxiety Inventory (STAI) [24, 25]. This questionnaire enables the quantification of current and habitual anxiety levels (20 items per scale) based on a 4-point Likert scale (score range: 0–60). Cronbach´s α values of .93 and .87 were reported for the State Anxiety and Trait Anxiety scales, respectively [25].
- Beck Depression Inventory (BDI) [26, 27]. This 21-item scale was used to quantify the severity of depression symptoms (4-point Likert scale, score range: 0–63). Cronbach´s α of the BDI is .95 [27].
- Fatigue Severity Scale (FSS) [28, 29]. This instrument assesses fatigue through 9 items (7-point Likert scale, score range: 9–63). It has a Cronbach´s α of .88 [29].
- Oviedo Quality of Sleep Questionnaire (COS) [30]. The Insomnia subscale of the COS was used, which includes 9 items (5-point Likert scale, score range: 4–54). The Cronbach´s α of this subscale is .88 [30].

### Attentional network test

**Fig 1:**
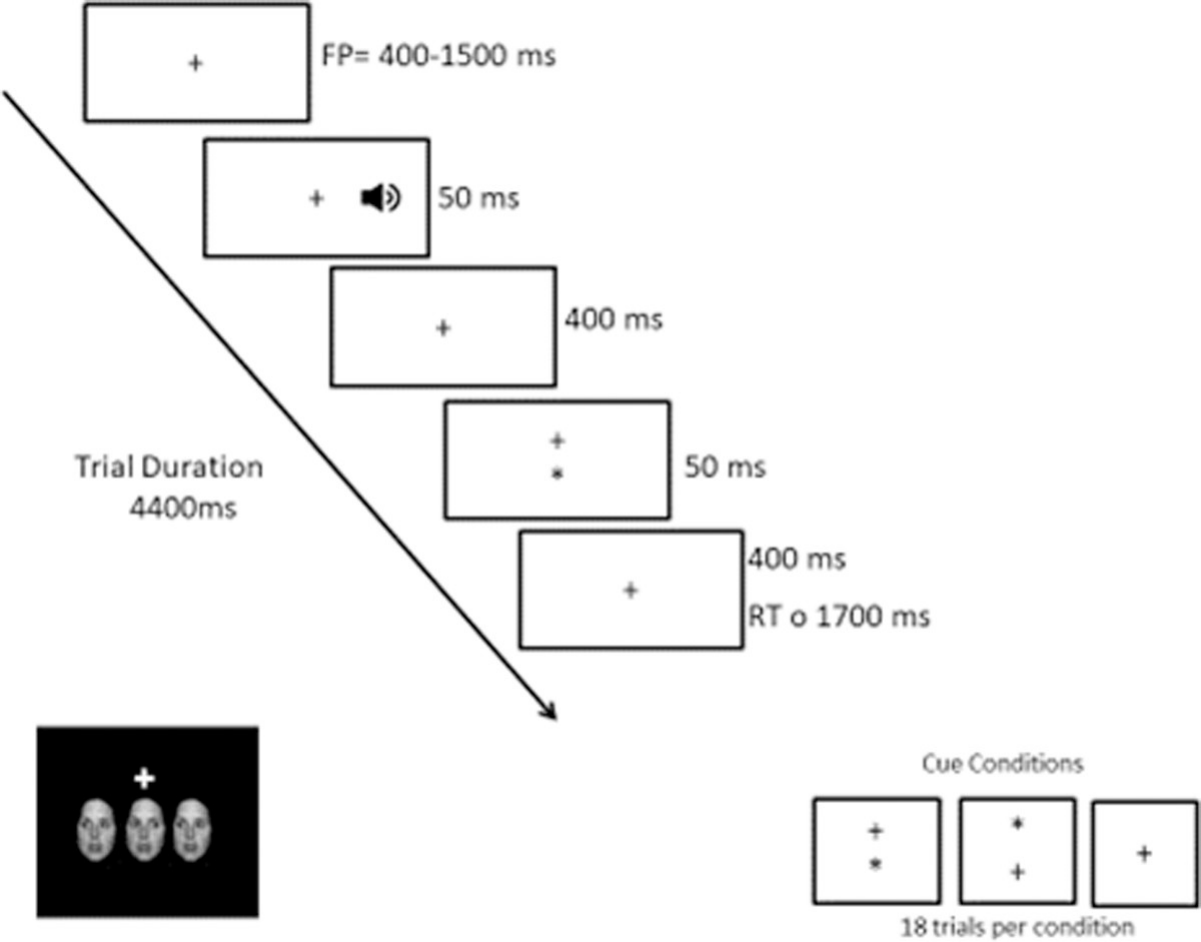
Sequence of events appearing in each trial of the ANT-I.

In the ANT-I, three faces appear in a row above or below a fixation cross; the participant has to indicate the gaze direction (right or left) of the central face (target stimulus) by pressing one of two keys. Three variables, representing the three attentional subsystems, were manipulated: alerting, cuing and congruency. The alerting variable consisted of a short tone presented, or not presented, before the appearance of the faces. The cuing variable had three levels, i.e. cued trials (asterisk presented at the same location as the subsequent target), uncued trials (asterisk presented in the location opposite to that of the subsequent target), and neutral trials (no asterisk). Congruency was manipulated by varying the gaze direction of the faces left and right of the target, corresponding (congruent trials) or not corresponding (incongruent trials) to the direction of the target. The task consisted of 4 blocks of 48 trials each, in which the 12 combinations of conditions (2 alerting x 2 cuing x 3 congruency) were equally represented. The timings of the task were as follows: fixation cross, 400–1500 ms; tone (if present), 50 ms; fixation cross, 400 ms; cue (if present), 50 ms; fixation cross, 400 ms; and faces, remained onscreen until response. Performance was indexed by reaction time (RT) and errors per condition. Fig 1 depicts the sequence of events appearing in each trial of the ANT-I.

### Procedure

The study was conducted in two sessions performed on the same day. During the first session, a clinical psychologist took the patients’ clinical history, recorded sociodemographic data and medication use, evaluated possible violations of the exclusion criteria, carried out the SCID interview and administered the self-report questionnaires. During the second session, the ANT-I task was performed. The test was presented on a desktop computer with a 15´ screen monitor using E-Prime software (version 1.2) [31]. Participants were asked not to consume analgesic drugs, alcohol, or caffeine, and not to practice rigorous physical exercise for 24 hours before the study. Written informed consent was obtained from all participants. The research accomplished all relevant regulations and institutional policies, was performed in accordance with the Helsinki Declaration and was approved by the Ethics Committee of the University of Jaén (Spain). The research data of the study is available to the public via the repository protocols.io (doi.org/10.17504/protocols.io.bq2zmyf6).

### Statistical analysis

To determine the optimal sample size based on expected effect sizes, the G*Power 3.1.7 program (University of Düsseldorf) was used. Previous comparisons between fibromyalgia patients and healthy individuals on attention tasks revealed medium to large effect sizes, with Cohen´s d ranging between 0.57 and 1.37 [2, 32]. Taking a conservative effect size of 0.52, an alpha level of 5% and a Beta error of 20% as a basis, a sample size of 40 participants per group appeared optimal.

Statistical analysis of RT was conducted using ANCOVAs performed separately for each attentional system. In each ANCOVA, group (FMS patients vs. RA patients vs. controls) served as the between-subjects factor. The experimental conditions of alerting (tone vs. no tone), cuing (cued vs. uncued vs. neutral) and congruency (congruent vs. incongruent) were the within-subject factors. Clinical pain severity was stronger in FMS than RA patients (see Table 1). Therefore, the Total Pain (MPQ) score was used as covariate in all models. Due to marginal group differences in years of education, this variable served as another covariate. The number of errors did not follow normal distribution (it was extremely left skewed, with 0 errors in most participants). Therefore, errors were compared between groups using the non-parametric Kruskal-Wallis test. Possible effects of medication use and comorbid depression and anxiety disorders on RT were evaluated by stratified analyses (ANCOVAs) in the patient sample (FMS and RA), comparing patients using and not using each type of medication (separately for antidepressants, anxiolytics, non-opioid analgesics, and opiates) and patients suffering and not suffering from depression and anxiety disorders. Effects of medication use and comorbid mental disorders on numbers of errors were tested through Mann-Whitney U tests. Effect sizes of the group comparisons in the ANT variables are indicated as Cohen´s d. Values of d between .2 and .5 correspond to small effect sizes, those between .5 and .8 indicate moderate effect sizes and those exceeding .8 represent high effect size [33]. Associations between questionnaire scores and ANT variables were computed by means of Pearson (for RT) and Spearman (for number of errors) correlations in both patient groups. Alpha was set at .05 in all analyses.

## Results

FMS patients exhibited higher Total Pain (MPQ), STAI State, STAI Trait, BDI, FSS and COS scores than RA patients and healthy controls (see Table 1). Moreover, RA patients had higher Total Pain, FSS and STAI State scores than controls. Depression, anxiety disorders, and the use of all assessed types of medications were more frequent in FMS patients than controls; depression and the use of anxiolytics and antidepressants were more frequent in FMS patients than RA patients; and anxiety disorders and the use of non-opioid analgesic and opiates were more frequent in RA patients than controls (see Table 1).

**Fig 2:**
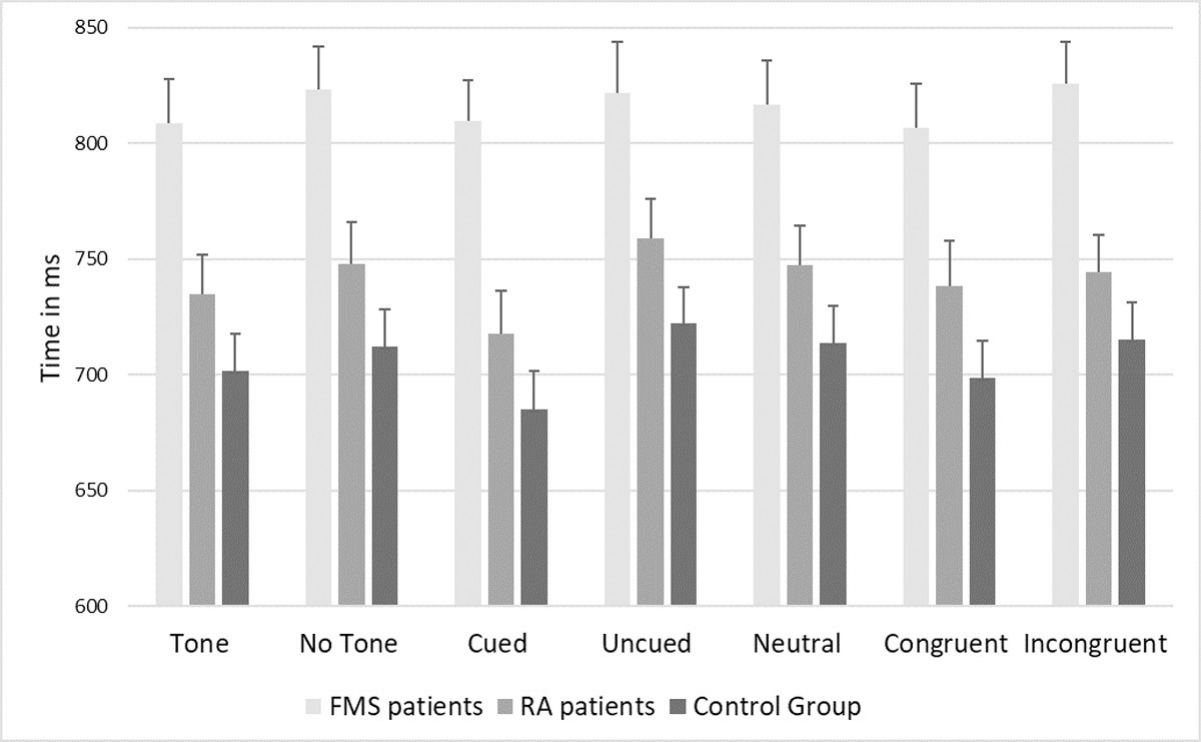
Reaction times in the Attentional Network Test (ANT-I) conditions of fibromyalgia syndrome (FMS) patients, rheumatoid arthritis (RA) patients and healthy controls. Note: Vertical bars represent standard errors of the mean.

Fig 2 displays the RTs during the ANT-I; in all conditions, RTs were longest in FMS patients, followed by RA patients and controls. The three ANCOVAs revealed main effects of group (alerting: F[2,142] = 8.75, p < .001, *ηp*^*2*^ = .11; cuing: F[2,142] = 8.75, p < .001, *ηp*^*2*^ = .11; congruency: F[2,142] = 8.75, p < .001, *ηp*^*2*^ = .11) and task condition (alerting: F[1,142] = 10.63, p = .001, *ηp*^*2*^ = .070; cuing: F[2,284] = 6.28, p < .01, *ηp*^*2*^ = .042; congruency: F[1,142] = 9.37, p < .01, *ηp*^*2*^ = .062). The interaction effect was not significant in any of the models. According to pairwise group comparisons, RTs differed between FMS and RA patients in all task conditions with small to medium effect sizes (all ps<0.01; Cohen´s d 0.021–0.60). While RT differences between FMS patients and controls were also significant with small to medium effect sizes (all ps<0.01; Cohen´s d 0.38–0.64), RTs did not differ between RA patients and controls (all ps >.01; Cohen´s d 0.019–0.47). Overall, RTs were shorter in the tone condition than in the no tone condition, and in the congruent condition than in the incongruent condition. Moreover, RTs were shorter in the cued condition than in the uncued and neutral conditions (all ps < .01, for all comparisons concerning the three networks; Cohen´s d 0.019–0.64). Table 2 presents the data on number of task errors. Kruskal-Wallis tests revealed more errors in FMS than RA patients in the cued (א^2^ = 4.22, p = .040, Cohen´s d = 0.25), neutral (א^2^ = 4.52, p = .033, Cohen´s d = 0.39) and congruent (א^2^ = 6.99, p < .01, Cohen´s d = 0.57) conditions with low to medium effect sizes. FMS patients made more errors than controls in the tone (א^2^ = 7.45, p < .01, Cohen´s d = 0.63), no tone (א^2^ = 4.65 p = .031, Cohen´s d = 0.33), cued (א^2^ = 4.70, p = .029, Cohen´s d = 0.47), neutral (א^2^ = 6.26, p = .012, Cohen´s d = 0.53), congruent (א^2^ = 5.83, p = .016, Cohen´s d = 0.44) and incongruent (א^2^ = 6.25, p = .012, Cohen´s d = 0.51) conditions with low to medium effect sizes. Finally, RA patients made more errors than controls in the uncued condition (א^2^ = 4.87, p = .027, Cohen´s d = 0.48) with a medium effect size.

**Table 2:** Numbers of errors (M±SD) on each condition of the ANT-I.

Task conditions	FMS patients (N = 56)	RA patients (N = 41)	Control group (N = 50)
**Tone trials**	0.31±0.39	0.19±0.29	0.12±0.15
**No Tone trials**	0.32±0.45	0.23±0.33	0.14±0.20
**Cued trials**	0.29±0.43	0.18±0.45	0.13±0.18
**Uncued trials**	0.26±0.53	0.25±0.38	0.11±0.18
**Neutral trials**	0.38±0.49	0.21±0.34	0.17±0.23
**Congruent trials**	0.23±0.30	0.09±0.14	0.12±.018
**Incongruent trials**	0.40±0.65	0.33±0.55	0.15±0.19

Neither psychiatric comorbidities (depression and anxiety disorders) nor medication use (antidepressants, anxiolytics, non-opioid analgesics and opiates) had a significant effect on RTs or numbers of errors (all ps>.05). Overall, correlations between questionnaire scores and the ANT variables were low. No correlations arose for RT in the seven experimental conditions. The number of errors in the neutral condition correlated positively with insomnia (COS) in FMS patients (r = .28, p = .039) and with Total Pain (MPQ) score in RA patients (r = .31, p = .046). The number of errors in the cued condition correlated negatively with depression scores (BDI) in RA patients (r = -.35, p = .025).

## Discussion

The longer RTs in FMS patients than RA patients and healthy controls in all ANT-I conditions confirm previous observations of attentional impairments in this disorder [2, 3]. This is also reflected by the findings pertaining to the number of errors; FMS patients made more errors than RA patients in 3 of the ANT-I conditions, and more errors than controls in 6 conditions of the task. The experimental conditions were designed to manipulate the attentional requirements in the domains of alertness, orienting and executive control. The magnitude of group differences did not vary by condition, which supports the notion of a more general deficit in attention and speed of information processing in FMS, rather than impairments in specific cognitive domains [34]. In contrast, RTs of RA patients did not exceed those of controls, and more errors in RA patients were only seen in one ANT-I condition. Importantly, clinical pain severity was controlled in the group comparison pertaining to RTs; as such, the greater impairments in FMS patients cannot be ascribed to greater pain experience. This finding contrasts with previous observations of cognitive decline of similar magnitude in RA and FMS patients [10–12]. The discrepancy may relate to differences in the task used and cognitive domains addressed, as well as to the relatively small sample sizes of earlier studies.

A possible hypothesis that may be tested in future studies is that central nervous sensitization to pain contributes to attention deficits in FMS [3, 14]. There is evidence that increased activity in the pain neuromatrix mediates the hyperalgesia and allodynia characterizing the disorder [13]. The brain networks involved in pain processing and attention partially overlap. Medial and lateral prefrontal areas and the anterior cingulate, for instance, are involved in nociception as well as attentional processing [13, 15]. Central nervous sensitization places more demands on these areas and thus reduces processing resources for attention. In a recent study using a dynamic evoked pain protocol, central nervous sensitization was far stronger in FMS than in RA [35]. Moreover, in FMS patients, allodynia resulting from central nervous sensitization is associated with cognitive impairments [36]. This supports the hypothesis that greater central nervous sensitization in FMS than RA is associated with more profound attentional impairments.

FMS patients showed a higher prevalence of comorbid depression and anxiety disorders and higher medication use than healthy controls [2, 34, 37]. Moreover, depression and the use of antidepressants and anxiolytics were more frequent in FMS patients than RA patients, whereas anxiety disorders and the use of non-opioid analgesics and opiates were more frequent in RA patients than controls. However, no effects of comorbid psychiatric disorders or medication on attentional performance were observed. Furthermore, no associations between the levels of clinical symptoms and RT variables were observed in the patients’ groups and associations concerning number of errors are scarce and not systematic.

The main limitation of the study refers to the control of medication use. Some relevant medications, in particular glucocorticoids in RA, were not assessed. Results pertaining to effects of glucocorticoid therapy on cognition remain controversial. Negative impact of glucocorticoids on memory [38] and hippocampal function [39] have been reported. However, other studies revealed a positive association between glucocorticoid medication and cognitive performance in RA [40]. Despite this limitation, this study confirmed the presence of a substantial general attention deficit in FMS. One possible hypothesis that may be tested in future studies is that lower attentional performance in FMS than RA reflect different pathogenetic mechanisms underlying the two disorders; in particular, the involvement of central nervous factors may be greater in FMS.

In summary, the study suggests of a more general deficit in attention and speed of information processing in FMS, rather than specific impairments in the domains of alerting, orienting and executive control.
